# The prevalence and risk factors of dyslipidemia in different diabetic progression stages among middle-aged and elderly populations in China

**DOI:** 10.1371/journal.pone.0205709

**Published:** 2018-10-16

**Authors:** Yaru Li, Liyun Zhao, Dongmei Yu, Gangqiang Ding

**Affiliations:** National Institute for Nutrition and Health, Chinese Center for Disease Control and Prevention, Beijing, China; Shanghai Diabetes Institute, CHINA

## Abstract

**Objectives:**

This study aimed to examine the prevalence and risk factors of dyslipidemia in different diabetic progression stages among middle-aged and elderly Chinese populations.

**Methods:**

The 2010–2012 China National Nutrition and Health Survey (CNNHS) is a nationally representative cross-sectional study. In the present study, a total of 69,974 participants aged ≥ 45 years were included. Dyslipidemia was defined based on the Chinese adult dyslipidemia prevention guide. A multivariable logistic regression model was performed to examine the associations between risk factors and dyslipidemia.

**Results:**

The prevalence of dyslipidemia was 39.9%, 46.8%, and 59.3% in participants with normal glucose, prediabetes, and type 2 diabetes mellitus (T2DM). Women had a lower dyslipidemia prevalence than men (38.7% vs. 43.3%). Dyslipidemia prevalence was positively associated with the education level and inversely correlated with the physical activity level, and negatively related to age only among prediabetes and T2DM groups (*P* for trend < 0.05). Obesity, abdominal obesity, and hypertension were associated with dyslipidemia.

**Conclusions:**

The prevalence of dyslipidemia was relatively high among middle-aged and elderly T2DM person. There are different associations between multiple risk factors and dyslipidemia in different diabetic progression stages.

## Introduction

The prevalence of type 2 diabetes mellitus (T2DM) has increased dramatically worldwide. According to the China Chronic Disease and Risk Factors Surveillance study, the estimated overall prevalence of diabetes and prediabetes was 10.9% and 35.7% among adults, respectively [1]. Individuals with T2DM had two-to-four fold increase in cardiovascular disease (CVD) risk [2]. Compared with healthy individuals, subjects with T2DM had an increased risk of cardiovascular mortality [3].

Dyslipidemia includes increased total cholesterol (TC), triglyceride (TG), and low-density lipoprotein cholesterol (LDL-C), and decreased high-density lipoprotein cholesterol (HDL-C) [4]. According to the China National Survey of Chronic Kidney Disease study, the prevalence of dyslipidemia was 34.0% among adults aged ≥ 18 years in 2010 [5]. A multicenter survey showed that the prevalence of dyslipidemia was 67.1% among T2DM subjects aged ≥ 40 years [6].

In 2015, about 290 million people had CVD. CVD is currently the number one cause of mortality in China and accounts for over 40% of total death [7]. Dyslipidemia and diabetes are all independent risk factors for CVD [8]. Compared with healthy individuals, subjects with dyslipidemia and diabetes had higher cardiovascular diseases risk [9]. CVD is still the leading cause of morbidity and mortality among T2DM patients, despite significant advances in management strategies to lessen CVD risk factors recently [10]. One major reason is atherogenic dyslipidemia. So early detection and effective management of dyslipidemia are urgent for reducing the rate of CVD morbidity and mortality, especially in diabetics [11].

Although several surveys showed that dyslipidemia prevalence increases among general populations, few studies have examined dyslipidemia prevalence and related factors in different diabetic progression stages. Therefore, we aimed to investigate dyslipidemia prevalence and associated factors in different diabetic progression stages among middle-aged and elderly populations based on the data of the 2010–2012 China National Nutrition and Health Survey.

## Methods

### Study population

The 2010–2012 China National Nutrition and Health Survey (CNNHS) is a nationally representative cross-sectional study on nutrition and non-communicable chronic diseases. This survey randomly selected 150 survey sites (districts or counties) of 31 provinces, autonomous regions, and municipalities directly. All survey sites were divided into four strata based on socioeconomic characteristics: large cities (34 survey sites), small-to-medium cities (41 survey sites), general rural areas (45 survey sites) and poor rural areas (30 survey sites). Six residential committees or villages were randomly selected from each survey site. And then 75 households were randomly selected from each residential committees or villages according to geographical location. Detailed information of this study has been described previously [12].

Individuals with missing data on T2DM, or serum lipid including TC, TG, LDL-C, and HDL-C were excluded. Participants with incomplete information on education level, smoking status, drinking status, and physical activity were further excluded. A total of 69,974 participants aged ≥ 45 years were included for the present study.

This survey was approved by the Ethical Committee of the National Institute for Nutrition and Food Safety, Chinese Center for Disease Control and Prevention. All participants provided written informed consent.

### Data collection

Height, fasting weight and waist circumference were measured with participants wearing light indoor clothing and no shoes. BMI was calculated as weight in kilograms divided by height in meters squared. Overweight was defined as a BMI between 24.0 and 28.0 kg/m^2^, and obesity was defined as BMI ≥ 28.0 kg/m^2^ [13]. Abdominal obesity was described as a waist circumference ≥ 90 cm for men and ≥ 85 cm for women according to guidelines for Chinese populations [14]. Blood pressure was measured three times after 5 minutes of rest, and the mean of the three measurements was used for analysis. Hypertension was defined as any of the following: systolic pressure ≥ 140 mmHg; diastolic pressure ≥ 90 mmHg; use of antihypertensive medications; or self-reported hypertension [15, 16].

Fasting plasma glucose, TC, TG, and HDL-C was measured by the hexokinase G-6- PDH method, the cholesterol oxidase-HMMPS method, the GPO-HMMPS glycerol blanking method, and the direct determination method, respectively. All measurements were conducted with Hitachi 7600 automated biochemical analyzer, and all reagents were produced by Wako Pure Chemical, Ltd. The LDL-C level was calculated using the Friedewald formula [17] (LDL-C = TC-HDL-C-TG/2.2) for the participants whose TG level < 4.52 mmol/L.

### Definition of dyslipidemia

According to the Chinese adult dyslipidemia prevention guide [14], dyslipidemia was defined as having any one of the following: high TC (serum TC level ≥ 6.22 mmol/L); high TG (serum TG level ≥ 2.26 mmol/L); high LDL-C (serum LDL-C level ≥ 4.14 mmol/L); low HDL-C (serum HDL-C level < 1.04mmol/L); use of antihyperlipidemic medications; or self-reported dyslipidemia.

### Assessment of prediabetes and T2DM

Prediabetes was defined as a participant suffering from impaired glucose tolerance (IGT) or impaired fasting glucose (IFG). According to the World Health Organization criteria [18], IGT was defined as fasting plasma glucose < 7.0 mmol/L and OGTT ≥ 7.8 and < 11.1 mmol/L. IFG was diagnosed with fasting plasma glucose 6.1–6.9 mmol/L and OGTT < 7.8 mmol/L. T2DM was diagnosed according to the American Diabetes Association criteria [19]: fasting plasma glucose ≥ 7.0 mmol/L; oral glucose tolerance test (OGTT) 2-h plasma glucose ≥ 11.1 mmol/L; hemoglobin A1c ≥ 6.5%; use of antidiabetic medications; or self-reported diabetes.

### Assessment of covariates

Trained investigators collected information including education level, smoking status, drinking status, and physical activity using a structured questionnaire. Education level was divided into uneducated, primary school, junior school, high school, and college or above. Smoking status was classified as never, ever, and current smokers. Drinking status was categorized into non-drinkers, moderate alcohol drinkers (with an alcohol intake of less than 175 g by men and 105 g by women per week), and excessive alcohol drinkers (with an alcohol intake of more than 175 g by men and 105 g by women per week). Physical activity level (PAL) was calculated according to the recommendation of the Institute of Medicine (IOM) [20] and was divided into quartiles.

### Statistical analysis

Normally distributed variables were expressed as means ± standard deviation (SD) and compared between groups using a z test. Skewed distributed variables were presented as medians (interquartile ranges) and compared between groups by non-parametric statistical hypothesis test. Categorical variables were expressed as number (percentages) and compared by the chi-square test. All results were weighted to represent the overall Chinese populations aged ≥ 45 years. Weight coefficients were obtained by calculating sampling weight and post-stratification weight. Sampling weight was computed based on the study design. Post-stratification weight was calculated based on the 2010 Chinese population census data. *P* value < 0.05 was considered statistical significance. All statistical analyses were conducted with SAS version 9.2 (SAS Institute).

## Results

The characteristics of the participants according to diabetic progression stages are presented in Table 1. The prevalence of prediabetes and T2DM was 13.2% and 11.6%. Subjects with prediabetes or T2DM were more likely to be older, urban residents, and with higher levels of TC, TG, LDL-C, waist circumference, BMI, and blood pressure, and lower level of HDL-C than those with normal glucose. The prevalence of dyslipidemia in normal, prediabetes, and T2DM group was 39.9%, 46.8%, and 59.3%, respectively. The rate of antihyperlipidemic medication taking is 7.6% for T2DM person.

**Table 1:** Characteristics of the participants according to diabetic progression stages (69974).

	Normal	Prediabetes	T2DM	*P*-value
N (%)	51546 (75.24)	9143 (13.17)	9285 (11.60)	
Age (years)	59.08 ± 9.37	61.41 ± 9.77	62.45 ± 9.41	< 0.001
Gender, n (%)				0.69
Men	22560 (49.51)	4045 (49.45)	4065 (48.44)	
Women	28986 (50.49)	5098 (50.55)	5220 (51.56)	
Area, n (%)				< 0.001
Urban	25769 (53.33)	4739 (49.35)	6196 (61.65)	
Rural	25777 (46.67)	4404 (50.65)	3089 (38.35)	
Education, n (%)				< 0.001
Uneducated	8396 (15.58)	1727 (19.96)	1437 (17.02)	
Primary school	17503 (33.67)	3140 (34.20)	2907 (32.00)	
Junior school	15951 (33.02)	2629 (29.80)	2783 (30.39)	
High school	7651 (14.79)	1245 (12.76)	1566 (15.66)	
College and above	2045 (2.95)	402 (3.27)	592 (4.93)	
Smoking, n (%)				< 0.001
Current smoker	13530 (29.82)	2276 (27.74)	1937 (23.52)	
Former smoker	2275 (4.08)	498 (4.94)	643 (5.67)	
Never smoker	35741 (66.10)	6369 (67.32)	6705 (70.82)	
Drinking, n (%)				< 0.001
Never drinker	36153 (66.64)	6435 (68.41)	6994 (74.16)	
Moderate alcohol drinker	10595 (21.77)	1754 (19.65)	1639 (17.56)	
Excessive alcohol drinker	4798 (11.59)	954 (11.94)	652 (8.28)	
Physical activity, n (%)				< 0.001
Low	12218 (23.43)	2474 (27.02)	2857 (31.05)	
Moderate	12285 (22.28)	2324 (23.72)	2709 (26.25)	
High	12929 (25.70)	2228 (24.31)	2440 (24.57)	
Very high	14114 (28.58)	2117 (24.95)	1279 (18.13)	
TC (mmol/L)	4.67 (4.09–5.30)	4.88 (4.26–5.53)	4.91 (4.27–5.57)	< 0.001
TG (mmol/L)	1.13 (0.80–1.64)	1.30 (0.88–1.91)	1.47 (1.02–2.16)	< 0.001
LDL-C (mmol/L)	2.85 (2.36–3.39)	3.00 (2.47–3.54)	3.01 (2.47–3.58)	< 0.001
HDL-C (mmol/L)	1.22 ± 0.33	1.20 ± 0.34	1.13 ± 0.31	< 0.001
Waist circumference (cm)	81.58 ± 9.84	84.32 ± 10.18	87.00 ± 10.04	< 0.001
BMI (kg/m^2^)	23.53 (21.36–25.90)	24.44 (22.09–26.93)	25.12 (22.93–27.46)	< 0.001
Systolic blood pressure (mmHg)	128.58 ± 20.93	133.20 ± 20.95	135.00 ± 21.89	< 0.001
Diastolic blood pressure (mmHg)	79.78 ± 11.76	81.40 ± 11.77	80.96 ± 12.22	< 0.001
Fasting plasma glucose (mmol/L)	5.09 (4.68–5.48)	6.18 (5.57–6.44)	7.27 (6.04–8.64)	< 0.001
Dyslipidemia, n (%)	21687 (39.85)	4489 (46.83)	5711 (59.28)	< 0.001
Antihyperlipidemic medication, n (%)	1223 (1.93)	271 (2.60)	824 (7.56)	< 0.001

Abbreviations: TC, total cholesterol; TG, triglyceride; LDL-C, low-density lipoprotein cholesterol; HDL-C, high-density lipoprotein cholesterol; BMI, body mass index; T2DM, type 2 diabetes mellitus.

Data are mean (standard deviation) for normally distributed or medians (interquartile ranges) for skewed parameters, or number (%).

Table 2 shows the characteristics of the participants according to dyslipidemia status. The overall prevalence of dyslipidemia was 43.0%. Participants with dyslipidemia were more likely to have prediabetes and diabetes than normal subjects.

**Table 2:** Characteristics of the participants according to dyslipidemia status (69974).

	Normal	Dyslipidemia	*P*-value
N (%)	38087 (56.98)	31887 (43.02)	
Age (years)	59.77 ± 9.67	59.90 ± 9.33	0.07
Gender, n (%)			< 0.001
Men	16100 (47.52)	14570 (51.83)	
Women	21987 (52.48)	17317 (48.17)	
Area, n (%)			< 0.001
Urban	19127 (51.61)	17577 (56.63)	
Rural	18960 (48.39)	14310 (43.37)	
Education, n (%)			< 0.001
Uneducated	6843 (17.84)	4717 (14.31)	
Primary school	13479 (35.18)	10071 (31.38)	
Junior school	11184 (31.30)	10179 (33.59)	
High school	5202 (13.04)	5260 (16.72)	
College and above	1379 (2.64)	1660 (3.99)	
Smoking, n (%)			< 0.001
Current smoker	9632 (28.34)	8111 (29.44)	
Former smoker	1624 (3.96)	1792 (4.93)	
Never smoker	26831 (67.69)	21984 (65.63)	
Drinking, n (%)			< 0.001
Never drinker	26587 (66.63)	22995 (69.23)	
Moderate alcohol drinker	7553 (21.11)	6435 (20.85)	
Excessive alcohol drinker	3947 (12.26)	2457 (9.92)	
Physical activity, n (%)			< 0.001
Low	8872 (22.75)	8677 (27.48)	
Moderate	8994 (21.53)	8324 (24.79)	
High	9417 (25.45)	8180 (25.31)	
Very high	10804 (30.27)	6706 (22.42)	
TC (mmol/L)	4.70 (4.19–5.22)	4.77 (4.04–5.72)	< 0.001
TG (mmol/L)	0.97 (0.72–1.31)	1.63 (1.12–2.38)	< 0.001
LDL-C (mmol/L)	2.85 (2.40–3.31)	2.95 (2.36–3.71)	< 0.001
HDL-C (mmol/L)	1.38 ± 0.26	1.00 ± 0.29	< 0.001
Waist circumference (cm)	80.20 ± 9.68	85.59 ± 9.80	< 0.001
BMI (kg/m^2^)	23.04 (20.93–25.36)	24.85 (22.68–27.09)	< 0.001
Systolic blood pressure (mmHg)	128.85 ± 21.04	131.45 ± 21.32	< 0.001
Diastolic blood pressure (mmHg)	79.39 ± 11.68	81.06 ± 11.96	< 0.001
Fasting plasma glucose (mmol/L)	5.20 (4.74–5.72)	5.38 (4.90–5.98)	< 0.001
Prediabetes, n (%)	4654 (12.29)	4489 (14.33)	< 0.001
T2DM, n (%)	3574 (8.29)	5711 (15.98)	< 0.001
Antihyperlipidemic medication, n (%)	—	2318 (6.22)	—

Abbreviations: TC, total cholesterol; TG, triglyceride; LDL-C, low-density lipoprotein cholesterol; HDL-C, high-density lipoprotein cholesterol; BMI, body mass index; T2DM, type 2 diabetes mellitus.

Data are mean (standard deviation) for normally distributed or medians (interquartile ranges) for skewed parameters, or number (%).

**Fig 1:**
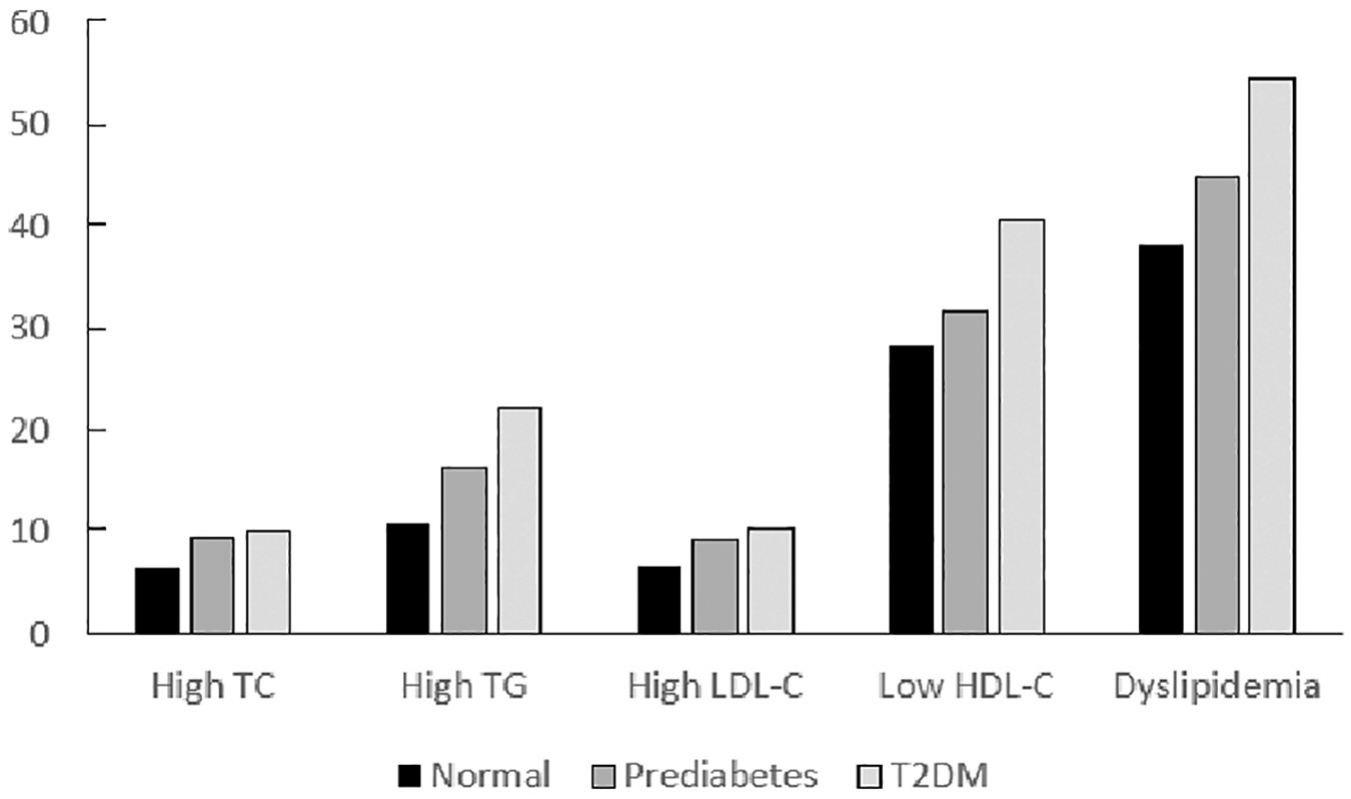
Prevalence of high TC, high TG, high LDL-C, low HDL-C, and dyslipidemia according to diabetic progression stages. Abbreviations: TC, total cholesterol; TG, triglyceride; LDL-C, low-density lipoprotein cholesterol; HDL-C, high-density lipoprotein cholesterol; T2DM, type 2 diabetes mellitus.

The prevalence of high TC, high TG, high LDL-C, low HDL-C, and dyslipidemia was 6.6%, 11.0%, 6.9%, 28.4%, and 39.9% among normal glucose subjects, 9.4%, 16.3%, 9.3%, 31.7%, and 46.8% among individuals with prediabetes, 10.3%, 22.3%, 10.3%, 40.8%, and 59.3% among T2DM person (Fig 1).

The associated factors of dyslipidemia in different diabetic progression stages are shown in Table 3. Women had a lower dyslipidemia prevalence than men in normal (OR: 0.75; 95% CI: 0.70–0.80) and T2DM (OR: 0.66; 95% CI: 0.56–0.77) groups. Dyslipidemia prevalence was positively associated with the education level and inversely correlated with the physical activity level, and negatively related to age only among prediabetes and T2DM groups (*P* for trend < 0.05). Smoking was associated with an increased dyslipidemia prevalence among the participants with normal glucose. Drinking was associated with a decreased prevalence of dyslipidemia. Subjects with obesity, abdominal obesity, and hypertension had a higher dyslipidemia prevalence than normal individuals.

**Table 3:** The associated factors of dyslipidemia in different diabetic progression stages (69974).

	Normal	Prediabetes	T2DM
Gender (women vs. men)	0.75 (0.70–0.80) ***	0.98 (0.80–1.21)	0.66 (0.56–0.77) ***
Area (rural vs. urban)	1.04 (0.84–1.30)	0.91 (0.67–1.24)	0.87 (0.68–1.13)
Age, years			
45–54	1.00 (ref)	1.00 (ref)	1.00 (ref)
55–64	0.99 (0.93–1.06)	1.04 (0.92–1.19)	0.90 (0.74–1.09)
65–74	0.96 (0.88–1.04)	0.84 (0.66–1.05)	0.83 (0.65–1.05)
≥75	0.88 (0.76–1.03)	0.72 (0.55–0.93) **	0.56 (0.38–0.82) **
*P*-trend	0.08	0.01	0.01
Education			
Uneducated	1.00 (ref)	1.00 (ref)	1.00 (ref)
Primary school	1.03 (0.92–1.15)	1.15 (0.95–1.39)	0.97 (0.77–1.23)
Junior school	1.12 (0.96–1.30)	1.47 (1.15–1.87) **	1.06 (0.82–1.38)
High school	1.28 (1.09–1.50) **	1.70 (1.17–2.47) **	1.17 (0.84–1.62)
College and above	1.33 (1.12–1.58) ***	1.76 (1.14–2.70) *	1.39 (0.94–2.06)
*P*-trend	< 0.001	< 0.001	0.04
Smoking			
Never smoker	1.00 (ref)	1.00 (ref)	1.00 (ref)
Current smoker	1.26 (1.16–1.37) ***	1.07 (0.91–1.26)	0.99 (0.85–1.17)
Former smoker	1.16 (1.03–1.31) **	1.13 (0.90–1.42)	1.02 (0.75–1.38)
Drinking			
Never drinker	1.00 (ref)	1.00 (ref)	1.00 (ref)
Moderate alcohol drinker	0.82 (0.75–0.89) ***	0.81 (0.69–0.95) **	0.80 (0.66–0.96) *
Excessive alcohol drinker	0.60 (0.53–0.67) ***	0.76 (0.60–0.96) *	0.59 (0.46–0.77) ***
Physical activity			
Low	1.00 (ref)	1.00 (ref)	1.00 (ref)
Moderate	0.96 (0.89–1.04)	0.98 (0.85–1.13)	1.09 (0.90–1.32)
High	0.88 (0.80–0.97) **	0.82 (0.69–0.97) *	0.82 (0.67–0.99) *
Very high	0.75 (0.68–0.83) ***	0.68 (0.55–0.84) ***	0.64 (0.52–0.79) ***
*P*-trend	< 0.001	< 0.001	< 0.001
Obesity (yes vs. no)	2.12 (1.76–2.54) ***	1.66 (1.40–1.97) ***	1.47 (1.28–1.70) ***
Abdominal obesity (yes vs. no)	1.54 (1.37–1.74) ***	1.76 (1.49–2.07) ***	1.52 (1.28–1.80) ***
Hypertension (yes vs. no)	1.20 (1.13–1.27) ***	1.24 (1.10–1.41) ***	1.47 (1.28–1.70) ***

Abbreviations: T2DM, type 2 diabetes mellitus; BMI, body mass index.

Adjusted for the sex, age, area, smoking status, drinking status, physical activity level, BMI, abdominal obesity, and hypertension. Except the variable of interest.

*: *P* < 0.05

**: *P* < 0.01

***: *P* < 0.001

Among T2DM person, high LDL-C was defined as serum LDL-C level ≥ 2.60 mmol/L. The prevalence of high LDL-C was 69.1% among participants without antihyperlipidemic medication and 66.8% among subjects taking antihyperlipidemic medications. Table 4 shows the associated factors of high LDL-C according to the presence or absence of antihyperlipidemic medications among T2DM. Women were more likely to have high LDL-C than men. High education level may be associated with an increased risk of abnormal LDL-C among participants without antihyperlipidemic medications, whereas related to a decreased risk of abnormal LDL-C among individuals with antihyperlipidemic medications.

**Table 4:** The associated factors of high LDL-C among T2DM (9285).

	Antihyperlipidemic medication	
	No (n = 8461)	Yes (824)
Gender (women vs. men)	1.69 (1.39–2.05) ***	1.93 (1.25–3.00) **
Area (rural vs. urban)	0.90 (0.65–1.26)	0.87 (0.45–1.69)
Age, years		
45–54	1.00 (ref)	1.00 (ref)
55–64	1.36 (1.13–1.63) ***	1.06 (0.59–1.90)
65–74	1.24 (0.99–1.57)	1.09 (0.51–2.35)
≥75	1.28 (0.97–1.69)	2.77 (0.95–8.06)
*P*-trend	0.03	0.19
Education		
Uneducated	1.00 (ref)	1.00 (ref)
Primary school	1.33 (1.02–1.74) *	0.63 (0.30–1.31)
Junior school	1.03 (0.77–1.37)	0.39 (0.17–0.91) *
High school	1.43 (1.06–1.94) *	0.65 (0.26–1.66)
College and above	1.43 (0.96–2.12)	0.44 (0.17–1.14)
*P*-trend	0.19	0.34
Smoking		
Never smoker	1.00 (ref)	1.00 (ref)
Current smoker	1.00 (0.84–1.19)	0.90 (0.44–1.87)
Former smoker	1.42 (1.05–1.93) *	0.97 (0.42–2.25)
Drinking		
Never drinker	1.00 (ref)	1.00 (ref)
Moderate alcohol drinker	1.31 (1.01–1.69) *	0.92 (0.52–1.62)
Excessive alcohol drinker	0.98 (0.73–1.33)	1.44 (0.43–4.83)
Physical activity		
Low	1.00 (ref)	1.00 (ref)
Moderate	1.13 (0.97–1.32)	1.43 (0.77–2.66)
High	1.01 (0.83–1.23)	0.97 (0.59–1.59)
Very high	1.09 (0.87–1.37)	0.67 (0.29–1.53)
*P*-trend	0.63	0.43
Obesity (yes vs. no)	1.34 (0.99–1.82)	0.70 (0.31–1.62)
Abdominal obesity (yes vs. no)	0.89 (0.75–1.06)	1.56 (0.77–3.15)
Hypertension (yes vs. no)	0.96 (0.80–1.15)	0.95 (0.41–2.16)

Abbreviations: BMI, body mass index.

Adjusted for the sex, age, area, smoking status, drinking status, physical activity level, BMI, abdominal obesity, and hypertension. Except the variable of interest.

*: *P* < 0.05

**: *P* < 0.01

***: *P* < 0.001

## Discussion

In the present study, we investigated the prevalence and risk factors of dyslipidemia in different diabetic progression stages among middle-aged and elderly populations in China. Our results showed that the prevalence of dyslipidemia was 39.9%, 46.8%, and 59.3% in normal, prediabetes, and T2DM subjects. Dyslipidemia prevalence was associated with age, sex, education level, smoking status, drinking status, physical activity level, and status of obesity, abdominal obesity, and hypertension. There existed different associations between multiple risk factors and dyslipidemia prevalence in different diabetic progression stages.

Our results showed that the prevalence of dyslipidemia was 43.0% among middle-aged and elderly populations in China. Data from the 2002 China National Nutrition and Health Survey showed that the prevalence of dyslipidemia was 18.6% among populations aged ≥ 18 years [21]. A cross-sectional study conducted among populations aged ≥ 35 years in 2007–2010, demonstrated that the overall prevalence of dyslipidemia was 34.0% [5]. The present study indicated that dyslipidemia prevalence has increased markedly over the past decades and highlights the urgent need to prevent and treat dyslipidemia in China.

The findings showed that the prevalence of dyslipidemia was higher in men (43.3%) than in women (38.7%), which is similar to previous studies [5, 21]. However, a cross-sectional study conducted among Chinese adults aged 45–89 years indicated that women had a higher dyslipidemia risk (OR:1.51; 95% CI: 1.25–1.83) [22] than men. Our findings also reported that the prevalence of high TG and low HDL-C was higher in men than in women, whereas the prevalence of high TC and high LDL-C was higher in women than in men. Previous studies also found the similar results [21, 23].

In the present study, the mean serum levels of TC, TG, LDL-C, and HDL-C among people with diabetes were 4.91, 1.47, 3.01, and 1.13 mmol/L. In China, the cutoff of serum lipid level for prevention of CVD in diabetics was: TC < 4.50 mmol/L, TG < 1.70 mmol/L, LDL-C < 2.60 mmol/L, and HDL-C > 1.04 mmol/L [14]. Among T2DM person, the prevalence of LDL-C abnormity (serum LDL-C level ≥ 4.14 mmol/L) was 10.3%; however, based on serum LDL-C level ≥ 2.60 mmol/L, the prevalence of LDL-C abnormity was 68.9%. Our results demonstrated that the control of dyslipidemia was unsatisfactory in T2DM patients, especially for the serum LDL-C level.

Our results showed that there exist different associations between multiple risk factors and dyslipidemia prevalence in the diabetic progression stage. Blood glucose metabolism is closely related to lipid metabolism [24]. A possible explanation for the difference between factors and dyslipidemia is that blood glucose metabolism may affect the relationship between these factors and dyslipidemia.

Dyslipidemia prevalence was negatively associated with age, which was inconsistent with other studies [22]. Further analyzed the association between dyslipidemia and age according to sex. We found that the prevalence of dyslipidemia was positively associated with age in women, but negatively associated with age in men. Also, another cross-sectional study also showed that older age was associated with an increased risk of dyslipidemia in women, whereas a decreased risk of dyslipidemia in men [25]. This might be due to that menopause is a significant risk factor of dyslipidemia in older women [26].

Higher education levels were associated with an increased risk of dyslipidemia prevalence. The possible explanation for this result is that participants with higher education levels may spend more time sitting in the office, have little time to exercise, frequently consume high-fat foods, and suffer from a work-related mental health problem. Further studies are needed to validate these findings and to investigate the potential mechanisms.

An exciting discovery is that the prevalence of low HDL-C significantly decreased among alcohol drinkers, which is similar to previous studies [23, 27]. Also, a survey conducted among middle-aged male showed that serum HDL-C level was higher in alcohol drinkers than in non-drinker [28]. The possible mechanisms involved in the association of alcohol intake and serum lipid levels are only partially understood [29]. Further prospective studies are needed to explore the association between alcohol consumption and low HDL-C risk.

This study was based on data from the CHNNS, which was conducted among participants from all 31 provinces, autonomous regions, and municipalities. Therefore, its findings may be seen as representative and convincible. However, several limitations should be considered. First, the prevalence of dyslipidemia was based on a single assessment of blood samples, which may lead to minor inaccuracies. Second, the cross-sectional design limits the ability to address causal relationships between associated factors and dyslipidemia. Third, because the sociodemographic information was obtained through a questionnaire, which may lead to recall bias. Fourth, serum LDL-C levels of participants were calculated with the Friedewald formula, which might underestimate LDL-C levels when TG levels are ≥150 mg/dl [30].

## Conclusion

In conclusion, the prevalence of dyslipidemia is relatively high among T2DM person. Age, sex, education level, smoking status, drinking status, physical activity level, BMI, waist circumference, and blood pressure are closely related to dyslipidemia. And there are different associations between multiple risk factors and dyslipidemia in different diabetic progression stages. These results highlight the urgent need for screening blood lipid levels and appropriate intervention programs, especially in T2DM person.

## Supporting information

S1 Table Analysis data. (XLSX)
